# Synthetic Health Data: Real Ethical Promise and Peril

**DOI:** 10.1002/hast.4911

**Published:** 2024-11-02

**Authors:** Daniel Susser, Daniel S. Schiff, Sara Gerke, Laura Y. Cabrera, I. Glenn Cohen, Megan Doerr, Jordan Harrod, Kristin Kostick‐Quenet, Jasmine McNealy, Michelle N. Meyer, W. Nicholson Price, Jennifer K. Wagner

**Keywords:** synthetic data, privacy, health data, machine learning, privacy‐enhancing technologies, research ethics, bioethics

## Abstract

Researchers and practitioners are increasingly using machine‐generated synthetic data as a tool for advancing health science and practice, by expanding access to health data while—potentially—mitigating privacy and related ethical concerns around data sharing. While using synthetic data in this way holds promise, we argue that it also raises significant ethical, legal, and policy concerns, including persistent privacy and security problems, accuracy and reliability issues, worries about fairness and bias, and new regulatory challenges. The virtue of synthetic data is often understood to be its detachment from the data subjects whose measurement data is used to generate it. However, we argue that addressing the ethical issues synthetic data raises might require bringing data subjects back into the picture, finding ways that researchers and data subjects can be more meaningfully engaged in the construction and evaluation of datasets and in the creation of institutional safeguards that promote responsible use.

## Essay

Modern health research and development faces a dilemma. On the one hand, there is more data than ever—in electronic health records, in lab research, in public datasets, and on the internet—from which to extract potentially transformative scientific insights and to use as the basis for developing breakthrough health care technologies. On the other hand, using this data entails various risks: threats to patient privacy, skewed samples and approaches to analysis that can perpetuate demographic and other biases, and uneven access to data about rare conditions and small patient subgroups. Generating synthetic data has emerged as one promising approach to potentially navigating these challenges.^1^

While there is no consensus definition of synthetic data, for present purposes, it is sufficient to understand synthetic data generation as a diverse suite of methods for creating datasets that are informative about real‐world phenomena—from economic trends to education outcomes to health risks—but contain little‐to‐no actually captured measurement data. Synthetic data is constructed to mirror the aggregate statistical properties of real‐world measurements;^2^ or, as a report from the Alan Turing Institute, the United Kingdom's national institute for data science and artificial intelligence (AI), describes it, it is “data that has been generated using a purpose‐built mathematical model or algorithm, with the aim of solving a (set of) data science task(s).”^3^ For example, synthetic data is being used to train machine‐learning (ML) systems (such as large language and computer vision models) and to test and validate new systems before real‐world deployment.^4^

A range of techniques for producing synthetic datasets are being explored, from simple approaches like masking and tokenizing (replacing potentially identifying values, such as names, with nonidentifying stand‐ins) to using AI/ML techniques to fabricate entirely artificial datasets that facilitate useful inferences about particular real‐world populations. In what follows, we focus primarily on this last category—machine‐generated synthetic data—which is used increasingly often in health care contexts. Under the hood, these efforts leverage the kind of generative AI technologies powering large language and image models, like OpenAI's ChatGPT and DALL‐E, respectively, to create datasets useful for scientific research and development. For example, synthetic x‐ray and computed tomography images have been used to train computer vision systems to recognize pathologies.^5^ Synthetic location data was used during the Covid‐19 pandemic to model disease spread.^6^ And researchers are developing methods for generating synthetic electronic health records to facilitate patient phenotyping^7^ and diagnosis forecasting.^8^

Because synthetic datasets are intended to contain little or no precise information about individual data subjects, they are viewed as helping to address concerns about patient privacy.^9^ Further, because they are constructed in a flexible fashion, they could—in theory—be designed to try to mitigate demographically biased samples or to otherwise augment or balance datasets for certain characteristics that may be scarce or difficult to obtain. In other words, synthetic data might be able to help fill data gaps (for rare conditions or small patient subgroups, for instance) so that limited datasets may still be used to train algorithms that power AI/ML diagnostic programs, predictive systems for medical screening, drug‐discovery tools, and related health technologies.^10^ Like so‐called digital twins—virtual models of real‐world systems (including, potentially, human patients) designed to enable testing of interventions by simulating their effects^11^—synthetic data is employed with the aim of increasing the speed and reducing the costs of science and engineering research.^12^

However, these approaches are still in their nascent stages, and their proposed benefits still need to be confirmed. Meanwhile, generating and using synthetic data also introduces risks and ethical trade‐offs that remain understudied. As the use of synthetic data grows, it is crucial that researchers, practitioners, and policy‐makers are clear‐eyed that, while synthetic data may help address certain ethical challenges facing users of health data, it is not a cure‐all for privacy concerns or a cost‐free solution for closing gaps in access to high‐quality data. In what follows, we outline possible benefits of synthetic data, as well as recurrent and emerging worries about privacy, fairness, and the reliability of synthetic data. We consider possible strategies for mitigating these concerns or balancing them against other values, and we discuss individual and institutional pathways for implementing such strategies.

## Potential Benefits and Risks of Synthetic Data

***Accuracy, reliability, and trust***. By definition, synthetic data is only an approximation of the measurement data it imitates. Thus, immediate questions arise about how good the approximation is: How valid are the statistical inferences drawn from any particular synthetic dataset? How reliable are methods of synthetic data generation (in general and in each specific case)? What are meaningful benchmarks? Is synthetic data more appropriate for certain uses or in certain contexts than others?

As synthetic data is used for a variety of purposes, it can be evaluated in different ways and along various dimensions.^13^ In some cases, one might be concerned about the fidelity of synthetic data—how closely it resembles measurement data.^14^ Perfect fidelity is neither possible nor desirable, however, as the goal of using synthetic data is often to minimize the privacy risks or biases in measurement data. Thus, in most cases, it will be more appropriate to gauge synthetic data's utility—how well it performs as a stand‐in for measurement data in relation to particular tasks.^15^ A number of strategies exist for evaluating the utility of individual synthetic datasets, as well as the underlying data‐generation methods themselves, including replication studies, assessment by domain experts, comparison with publicly available aggregate data, and testing against general utility metrics.^16^ Yet, as Richard Chen and colleagues point out, in health contexts, especially involving rare or understudied conditions, there might be too little measurement data to develop benchmarks or clinical reference standards.^17^ Further, these standards are still in flux and lack consensus among the scientific community, though efforts to define them are under way in both the United States and the European Union.^18^

Perceptions of accuracy and reliability are likely to impact trust in and acceptance of these technologies. While synthetic data could help improve trust in health science by making data more widely available and the results of data analysis more reproducible, careless adoption of synthetic data practices could undermine trust and lead to doubts about reliability.^19^ Moreover, as with AI technologies in general, the incorporation of synthetic data‐driven tools into clinical practice could affect patient trust in doctors and other health care providers.^20^ As we discuss below, emphasis should be placed on making these technologies trust*worthy* by developing institutional structures that foster accountability and legal and regulatory frameworks that support these structures.^21^

***Privacy, security, and regulatory oversight***. Proponents of using synthetic data in health research point to a range of possible benefits, chief among them privacy, security, and wider access to otherwise sensitive health information.^22^ Synthetic data could offer privacy advantages over traditional “data sanitization” methods, such as simple deidentification or anonymization, by further reducing the statistical likelihood of reidentifying real patients—especially when used in combination with other privacy‐enhancing methods such as differential privacy (which involves adding calibrated statistical noise to a dataset or results derived from it with the goal of preventing the disclosure of information about specific individuals while preserving important distributional features and providing useful statistical information).^23^ It could also help researchers avoid cybersecurity threats by minimizing the amount of real patient data they need to store and that is therefore susceptible to unauthorized access.^24^

More broadly, researchers are enthusiastic about the potential for synthetic data to widen access to information in health science and scientific training. Like digital twins, synthetic data could enable researchers to safely explore health data before testing hypotheses with real measurement data, and it could offer opportunities for educating and training data scientists and other health care practitioners and researchers, allowing them to gain experience while minimizing the exposure of real patient data.^25^ It could, in theory, foster more reproducible data science by assuaging worries about sharing data after research has been conducted.^26^ And it could help democratize health research and development in general by increasing access to data that is currently off‐limits for privacy or intellectual‐property reasons.

- **Data leakage and adversarial attacks**. However, while synthetic data credibly promises to ease some worries about privacy and security in health data, it also has meaningful limitations. First, reidentification of individual data subjects from synthetic data may be more difficult, but it is not impossible. Depending on how synthetic datasets are constructed, they may leak more or less information about the actual measurement data they mimic.^27^ Partially synthetic data still contains some true measurement data that could be used to reidentify records. For example, researchers have developed systems for generating partially synthetic versions of clinical notes from electronic health records, containing a mix of real note text and artificially generated text.^28^ Moreover, even fully synthetic datasets created using AI/ML methods can sometimes overfit the measurement datasets they are designed to simulate—mirror them too closely, that is—potentially revealing underlying data, such as distributions for small subgroups that can be used for population‐ or individual‐level inferences.^29^ The United Kingdom's Office of National Statistics describes a “synthetic dataset spectrum” from, on one end, “structural synthetic datasets” that mirror only the overarching structures of measurement datasets (the types and names of variables they contain, but no true values or statistical relationships) to, on the other end, “replica datasets” that mirror many of the real relationships (joint distributions) between measurement data.^30^ Datasets derived from methods at the latter end of the spectrum represent higher disclosure risks. Whether through direct inference of underlying data or the use of proxy variables, in isolation or in combination with other data sources, synthetic data thus does not eliminate all risks of disclosure, even when combined with techniques like differential privacy.^31^
- **Group harms**. Second, like other privacy‐preserving technologies—such as anonymization (removing identifying information from a dataset or making sensitive information in the dataset less specific) and differential privacy—synthetic data is designed, to the greatest extent possible, to minimize disclosure of information about individuals while facilitating population‐level inferences and statistical analyses. But, as privacy scholars point out, individuals can nevertheless be harmed even by aggregate statistics, which may reveal sensitive information about groups to which they belong and enable exploitation of this information.^32^ For instance, a life insurer might erroneously raise someone's rates simply because it believes incorrectly that the individual is statistically similar to people who have developed dangerous health conditions.^33^ Completely eliminating this problem may not be feasible—population‐level statistics are the basic building blocks of health and human science.^34^ Still, scientists need to recognize the potential group harms and ensure that the statistics they generate are not produced or shared haphazardly and are protected adequately in light of possible harms of disclosure or misuse.
- **Circumventing regulation**. Third, despite the limitations described above, synthetic data might not be considered “personally identifiable information” or “protected health information” under the U.S. Health Insurance Portability and Accountability Act (HIPAA) and therefore might be exempt from legal constraints on data use and sharing.^35^ Scholars appear to disagree as to whether data‐protection laws do or even should apply to synthetic data.^36^ Much of this will likely depend on the synthetic data in question. For example, if the synthetic data is sufficiently nonidentifiable, perhaps it should fall outside of the information protected by HIPAA to accelerate scientific understanding of human health.

***Bias, fairness, and justice***. Though synthetic data has shown promise in certain contexts, researchers ought to be sensitive to its failure modes (why and how the use of synthetic data can go wrong) and—crucially—who is most likely to suffer the consequences of its failure. On the one hand, proponents of synthetic data tout its potential for correcting problematic biases in data‐driven health research. Synthetic data and related simulation technologies can be used to surface and explore biases in data‐generation mechanisms, helping researchers better understand the sources and impacts of bias.^37^ In addition, where bias stems from the underrepresentation of minorities or other groups in measurement datasets (often due to disparities in the recruitment and selection of data subjects in clinical trials), synthetic data could, in theory, be used to fill data gaps and thereby balance representation in datasets.^38^

On the other hand, here again, it's important to tread carefully. While judicious applications of synthetic data could plausibly help address these problems in certain contexts, synthetic data can also introduce new pathways for bias and unfairness. The rapidly growing field of fair AI/ML has demonstrated the harms and persistence of bias in datasets, algorithms, models, and real‐world applications of AI systems, as well as the challenges of formally defining fairness across social contexts.^39^ Researchers have found that commonly used synthetic data generation tools (such as HealthGAN) can produce datasets in which the fidelity or resemblance of synthetic health data to measurement data differs across sociodemographic groups.^40^ And applying differential privacy protections to synthetic data (strengthening its privacy guarantees) can lead to unfairness because adding noise to datasets produces uneven effects for different subgroups represented in the data. Recent empirical findings have shown, for example, that using differentially private synthetic data to train machine‐learning models can lead to differences in the influence of majority versus minority subgroups on downstream classifications, which suggests the normative trade‐offs researchers will continue to have to manage.^41^

Furthermore, the use of synthetic data might raise fairness and justice concerns beyond issues of bias. For example, as with other big data‐driven research, synthetic data‐generation methods that start with real‐world patient data raise difficult questions about arguably incomplete forms of consent.^42^ Moreover, health researchers have long wrestled with whether and how to fairly compensate clinical research subjects for participating in studies and how to share the benefits derived from their data—an issue that remains highly controversial.^43^ Such questions are becoming even more complex as biomedical research relies increasingly on big data resources that pool together data from large numbers of data subjects. One driver of these problems is the disconnection of patients from data about them—the “severing of the relationship between patients and their data.”^44^ Some commentators believe that there is a duty to share health care data under certain conditions.^45^ Insofar as synthetic data promises to further distance datasets from the real‐world phenomena they capture, it could be argued that synthetic data reduces rather than increases the argument for compensation.

Another potential concern from a fairness perspective is that researchers might turn to synthetic data in lieu of investing in the important community engagement work needed to collect diverse real‐world data and set a foundation for trusted relationships, mutual understanding, and sustainable support for the research to be conducted responsibly and effectively. Researchers could, for example, be especially incentivized to rely on synthetic data when capturing data from hard‐to‐reach populations is costly. While efforts to mitigate bias without burdening minority communities are laudable, careful and direct engagement is especially important in these cases, and—from a technical perspective—diverse measurement data is necessary to fully address fairness concerns.^46^ If these issues are not weighed carefully, the use of synthetic data as a replacement for measurement data could not only further marginalize the needs, interests, and priorities of those communities but also lead to both an underappreciation of the true range of human variation and an overreliance on the limited range of diversity reflected in current research datasets.

## Navigating the Ethics of Synthetic Data

In short, although using synthetic data as a replacement for measurement data could help address some ethical concerns, it raises others. Familiar issues encountered when using measurement data persist in applications of synthetic data, and synthetic data can introduce new problems of its own. Approaching synthetic data as a remedy for privacy, fairness, and related problems requires grappling carefully with their underlying causes.

Generally, the motivation for using synthetic data has been to “take the people out”—that is, to create or modify datasets in such a way that, although the resulting datasets are representative of real people or real‐world phenomena, negative and disparate impacts of measurement data and the inferences made from such data are removed. Although, in some instances, that may be the most privacy‐protective approach, there are some contexts where it is not the ideal strategy. In some instances, the goal should instead be to find ways to *bring people back in*: to find ways that people, both researchers and data subjects, can be more meaningfully engaged in the construction and evaluation of datasets and in the creation of institutional safeguards that promote responsible use.^47^ We envision the facilitation of such engagement in (at least) two ways: building institutional structures that foster accountability in educational, research, and health care settings and developing policy frameworks that give those structures weight and force.

At a minimum, as synthetic data techniques are more and more frequently incorporated into health care research and practice, educational institutions can reflect those changes in the way they train new researchers and clinicians. Disciplines that are training students to create and use synthetic data should introduce and require coursework emphasizing the benefits, risks, and broader ethical and societal implications of synthetic data techniques. Universities and accreditation bodies should put their institutional heft behind such efforts, perhaps including them in program requirements.

As necessary as ethics awareness and education are, however, they are not sufficient for ensuring ethical data practices on the ground. Health, social science, and other research involving human subjects has a history of systems of review and assessment, including institutional review boards (IRBs), risk assessment agencies, and independent ethics committees. In addition, at the beginning and end of the research process, funders and scientific journals, editorial boards, and peer reviewers, respectively, may have roles to play in ensuring that synthetic data is used ethically and responsibly. Many of these institutional structures and the intraorganizational nodes that reflect them were created in response to highly discriminatory and harmful experimental and data collection methods. Because synthetic data is a relatively new tool, there is the possibility of leveraging and enhancing these existing systems to anticipate and avert the negative impacts outlined above.

If these boards, agencies, and committees weigh in on the ethics of synthetic data, their members will themselves need to be educated about the risks, benefits, and trade‐offs involved in using synthetic data versus its alternatives. For this reason and others, the proposal to involve these groups may meet with some criticism. First, it is unclear whether the creation or use of synthetic data constitutes human subjects research under the Common Rule (the federal regulation governing research on human subjects), and such foundational decisions affect the extent to which IRBs are authorized to act and whether IRB action would be overreaching mission creep or legitimate oversight. It is also unclear whether action on synthetic data would be a wise use of oversight resources.

Beyond these familiar approaches, researchers and their institutions can find new ways to bring people into decision‐making about when and how to use synthetic data. Organizations using synthetic data could involve communities likely to be affected by it in their policy‐making processes, both to help critically assess and surface potential impacts and to design enforcement mechanisms that could repair trust after failures in compliance. One of the biggest challenges to incorporating this suggestion, though, might be identifying the communities that will potentially be impacted by the use of synthetic data. Once they are identified, community preview strategies and anticipated impact statements might be adopted, whereby proposals to create or use synthetic data would be submitted for review at the ideation stage to identify problems that might arise with the data and their use and to correct for them in advance. And community auditing processes could be used to catch problems that emerge after dataset creation.^48^ Review boards and open‐source research communities are wellsprings of experience that organizations could look to for help in developing and implementing these practices.

Finally, there could be an important role for law and regulation to play in clarifying or buttressing these strategies—in the form of either new legislation or updates to existing rules, such as HIPAA; the Federal Food, Drug, and Cosmetic Act; and the Common Rule. Federal offices and agencies like the U.S. Department of Health and Human Services’ Office of Civil Rights, Office for Human Research Protections, and Food and Drug Administration; the Federal Trade Commission; and the Office of the National Coordinator for Health Information Technology could solicit comments and issue guidance on appropriate use of synthetic data techniques, while standards bodies could incorporate considerations about synthetic data into new and updated standards efforts.^49^ These offices, agencies, and standards bodies could define expectations for research institutions, companies, and health care providers in the treatment of and transparency around synthetic data; could develop best practices for dataset creation and evaluation; and could perhaps even create public tools and infrastructure to facilitate safer synthetic data production and research.

## Acknowledgments and Disclaimer

We appreciate the opportunity provided by Alex Bui to pursue this bioethical work as a supplement to the PREMIERE study (A Predictive Model Index and Exchange Repository, supported by grant no. 3R01EB027650‐03S1 from the National Institutes of Health's Office of the Director and the National Institute of Biomedical Imaging and Bioengineering) and for input from Anders Garlid on early discussions that influenced the manuscript's development. Sara Gerke and Jennifer K. Wagner report grant no. 1R21EB035474‐01 from the NIH Office of the Director and NIBIB. The content is solely the responsibility of the authors and does not necessarily represent the official views of the NIH. Sara Gerke also reports grants from the European Union (grant agreement no. 101057321 and no. 101057099). Glenn Cohen reports a Novo Nordisk Foundation grant (no. NNF23SA0087056) for a scientifically independent International Collaborative Bioscience Innovation & Law Programme.
